# Investigation of Tribological Characteristics of PEO Coatings Formed on Ti6Al4V Titanium Alloy in Electrolytes with Graphene Oxide Additives

**DOI:** 10.3390/ma16113928

**Published:** 2023-05-24

**Authors:** Sergey Grigoriev, Nikita Peretyagin, Andrey Apelfeld, Anton Smirnov, Alexei Morozov, Elena Torskaya, Marina Volosova, Oleg Yanushevich, Nikolay Yarygin, Natella Krikheli, Pavel Peretyagin

**Affiliations:** 1 Spark Plasma Sintering Research Laboratory, Moscow State University of Technology “STANKIN”, Vadkovsky per.1, Moscow 127055, Russia; s.grigoriev@stankin.ru (S.G.); n.peretyagin@stankin.ru (N.P.); m.volosova@stankin.ru (M.V.); 2 Scientific Department, A.I. Evdokimov Moscow State University of Medicine and Dentistry, Delegatskaya St., 20, p.1, Moscow 127473, Russia; olegyanushevich@mail.ru (O.Y.); jarigin@msmsu.ru (N.Y.); nataly0088@mail.ru (N.K.); 3 Department 1203, Moscow Aviation Institute, National Research University, Volokolamskoe Shosse, 4, Moscow 125993, Russia; apelfeld@yandex.ru; 4 Laboratory of Tribology, Ishlinsky Institute for Problems in Mechanics RAS, pr. Vernandskogo, 101-1, Moscow 119526, Russia; morozovalexei@mail.ru (A.M.); torskaya@mail.ru (E.T.)

**Keywords:** Ti6Al4V titanium alloy, plasma electrolytic oxidation, graphene oxide, PEO coating, surface morphology, friction, wear

## Abstract

Coatings with a thickness from ~40 to ~50 µm on Ti6Al4V titanium alloys were formed by plasma electrolytic oxidation (PEO) in a silicate-hypophosphite electrolyte with the addition of graphene oxide. The PEO treatment was carried out in the anode–cathode mode (50 Hz) at a ratio of anode and cathode currents of 1:1; their sum density was 20 A/dm^2^, and the treatment’s duration was 30 min. The effect of the graphene oxide’s concentration in the electrolyte on the thickness, roughness, hardness, surface morphology, structure, composition, and tribological characteristics of the PEO coatings was studied. Wear experiments, under dry conditions, were carried out in a ball-on-disk tribotester with an applied load of 5 N, a sliding speed of 0.1 m·s^−1^, and a sliding distance of 1000 m. According to the obtained results, the addition of graphene oxide (GO) into the base silicate-hypophosphite electrolyte leads to a slight decrease in the coefficient of friction (from 0.73 to 0.69) and a reduction in the wear rate by more than 1.5 times (from 8.04 to 5.2 mm^3^/N·m), with an increase in the GO’s concentration from 0 to 0.5 kg/m^3^, respectively. This occurs due to the formation of a GO-containing lubricating tribolayer upon contact with the coating of the counter-body in the friction pair. Delamination of the coatings during wear occurs due to contact fatigue; with an increase in the concentration of GO in the electrolyte from 0 to 0.5 kg/m^3^, this process slows down by more than four times.

## 1. Introduction

Titanium alloys, in particular Ti6Al4V, which are valve metals, have found wide application in various industries, such as automotive [1], aerospace [2], oil [3], biomedical [4], etc., due to their low density, high specific strength, and fatigue resistance. However, the unsatisfactory hardness [5] and wear resistance characteristics [6] of Ti6Al4V titanium alloys still limit its application [7]. Therefore, new reinforcing phases are needed to improve the characteristics of titanium alloys. The discovery of graphene [8], with its excellent mechanical and physical properties, has provided new opportunities for the development of modern composites. It has been pointed out that graphitic structures are able to significantly improve most of the properties of Ti6Al4V alloys. In [9], by the method of spark plasma sintering (SPS), a Ti6Al4V composite reinforced with graphene oxide (GO) nanosheets was obtained and demonstrated a better balance of strength (tensile strength of 1041.1 MPa) and plasticity (yield strength of 921.8 MPa and elongation of 5.3%) than the composite with a 0.54 wt.% of GO when adding a 0.27 wt.% of GO. An increase in the strength characteristics was also noted in [10], in which the graphene network (GN)-reinforced Ti6Al4V composites were obtained by powder metallurgy. The results showed that the yield strength and the strength of the composite GN@Ti6Al4V with a 0.1 wt.% of graphene increased by 18.72% and 9.31%, respectively, as well as an improvement in the plasticity of the composite by 8.66%. The addition of a 0.5 wt.% of GO to the surface of Ti6Al4V (Ti64) powders by electrostatic self-assembly in work [11] demonstrated an increase in the yield strength from 1375 MPa to 1793 MPa and an increase in compressive strength from 1796 MPa to 2032 MPa for GO/Ti64 samples made by laser powder bed deposition (L-PBF). In [12], the authors’ laser cladding on Ti6Al4V alloys produced composite coatings containing a 0.5 wt.% of graphene oxide and determined an increase in microhardness of about 80 HV0.2, as well as an increase in the corrosion resistance of the coating at a laser power of 1600 W. Studies of the coatings in [13], including graphene oxide, reduced graphene oxide, TiC, and TiB2 obtained on Ti6Al4V alloys by electroerosion-coating, showed that the coefficient of friction (COF) was reduced by about 43%, the hardness was increased 3.9 times, and the linear corrosion resistance was increased by 19 times. The deposition of graphene oxide on the surface of Ti6Al4V titanium alloy filaments using laser-induced breakdown spectroscopy (LIBS) was presented in a study [14] in which it was demonstrated that graphene oxide has the ability to integrate into the structure of the filament surface, thereby increasing biocompatibility and osseointegration. The effect of reduced graphene oxide (rGO), which is part of the coatings based on hydroxyapatite (HA) obtained by the sol–gel method, on the corrosion resistance and cell engraftment of the porous alloy, Ti6Al4V (P-Ti6Al4V), was presented in [15]. The results showed a decreased corrosion rate and improved cell viability, in vitro, for samples with a 0.5 and a 1.0 wt.% of rGO-HA. The authors of [16] showed an increase in the antibacterial properties of Ti6Al4V alloys with the electrodeposition of the composite coating of graphene oxide (GO) and strontium (Sr) on its surface. In [17], the graphene oxide/carbon fiber/polyetheretherketone (GO/CF/PEEK) composite coatings obtained by electrostatic powder deposition on Ti6Al4V alloys demonstrated a decrease in the friction coefficient (the COF) (from 0.433 to 0.008), increased wear resistance, and good cytocompatibility. Nanoscale graphene oxide coatings obtained by laser texturing followed by electrophoretic deposition on Ti6Al4V alloys improved biocompatibility by increasing cell adhesion and proliferation [18]. In addition to the development of metal matrix composites, Ti6Al4V-graphene, there is an alternative method for improving the characteristics of titanium-based alloys. Plasma electro-oxidation (PEO), also known as micro-arc oxidation (MAO), is a well-established surface layer modification method that uses the build-up of a ceramic-like coating on the surface of valve metals (Al, Mg [19], Zr [20], and Ti [21]). The resulting PEO coating improves such material properties as wear resistance [22], corrosion protection properties [23], dielectrics, thermal protection, and decorative characteristics [24]. The composition of the electrolyte, which can contain various micro- and nanoscale additives [25,26], is important for obtaining the desired improvement in the properties of the surface layers [27]. The most promising are PEO coatings with embedded carbon-containing additives, such as graphite [28,29], carbon nanotubes (CNTs) [30], graphene of various shapes [31], and graphene oxide (GO) [32]. GO in the composition of the PEO coating has a favorable effect on mechanical and tribological characteristics due to the filling and sealing of the micropores [33]. Wang et al. [28,29] studied duplex PEO coatings with sprayed graphite obtained on Ti6Al4V substrates in NaAlO_2_-based electrolytes. These coatings had high adhesion to the alloy and antifriction properties. Guo et al. [30] analyzed the effect of CNT addition on the tribocorrosive properties of PEO coatings on Ti6Al4V alloys. The concentration of CNTs in the base electrolyte solution was 0.05, 0.1, 0.15, and 0.2 kg/m^3^. It was discovered that MAO coatings with 0.15 kg/m^3^ CNTs had the best tribocorrosion resistance, the lowest wear rate, and a high hardness with low porosity and surface roughness. Chen et al. [31] studied the friction and wear of PEO coatings on TC4 alloys with different graphene contents (0 kg/m^3^, 0.5 kg/m^3^, 3 kg/m^3^, and 6 kg/m^3^) in electrolytes. As a result, it was found that the main components of the coating were TiO_2_, amorphous materials, and SiC formed by graphene during the reaction, as well as graphene particles entering the film by physical adsorption. Friction and wear tests showed that the PEO coatings had low wear compared to the titanium matrix. Mazinani et al. [32] investigated the effect of the incorporation of GO into porous PEO titanium surfaces on antibacterial behavior. The electrolyte consisted of ethylene glycol, 94.5 wt%, deionized water, 5 wt%, and potassium fluoride, 0.5 wt%, to which 0.5 mg of GO in the form of suspension was added. Besides enhanced bioproperties, the results showed that the microhardness (171 HV) of the PEO coating formed in the electrolyte with the GO addition was increased compared to the coating obtained in the electrolyte without such an additive. Zhang et al. [33] obtained graphene-containing PEO coatings with a graphene content of 0, 0.05, 0.10, 0.15, and 0.20 kg/m^3^ on the titanium alloy Ti-5Al-1V-1Sn-1Zr-0.8Mo in an electrolyte containing 16 kg/m^3^ of Na_2_SiO_3_, 2.0 kg/m^3^ of Na_2_EDTA, and 10.0 kg/m^3^ of Na_2_HPO_4_. The addition of graphene reduced the coatings’ roughness and the size of the pores and microcracks and increased the wear resistance.

In most of the studies on carbon-containing PEO coatings on titanium alloys, the PEO treatment was performed in the anodic mode (or the mode was not specified at all), but the properties of the PEO coatings with GO performed in the anode–cathode mode were not analyzed. The aim of this study is to analyze the effect of GO additives in the silicate-hypophosphite electrolyte on the surface morphology, friction, and wear properties of coatings formed by plasma electrolytic oxidation in the anode–cathode mode on the Ti6Al4V titanium alloy.

## 2. Materials and Methods

**Figure 1:**
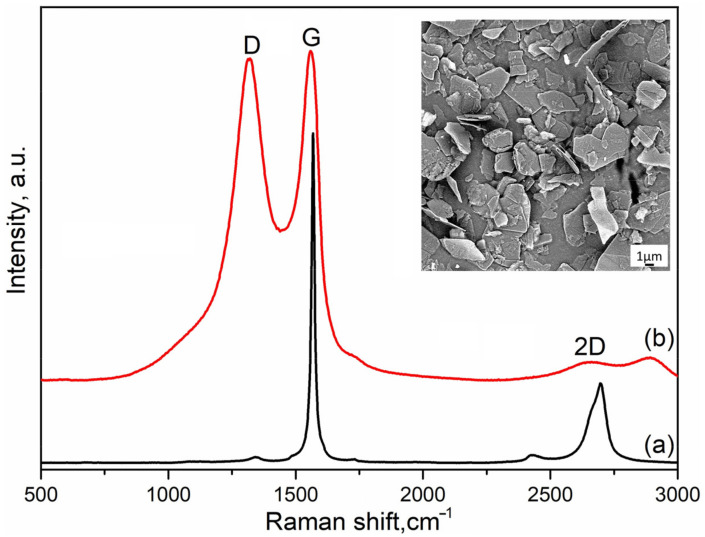
SEM image and Raman spectra of raw graphite powder (**a**) and produced graphene oxide (**b**).

Graphene oxide (GO) was obtained by the modified Hammers method, a detailed description of which is presented in the previous papers [23,24,25,26,27,28,29,30,31,32,33,34,35]. Commercial graphite powder (Plasmotherm, Moscow, Russia) with a median particle size of d_50_ = 3 μm was used for GO fabrication. An SEM image of the initial graphite powder and the typical characteristic Raman’s peaks of the graphite and graphene oxide is shown in Figure 1.

**Figure 2:**
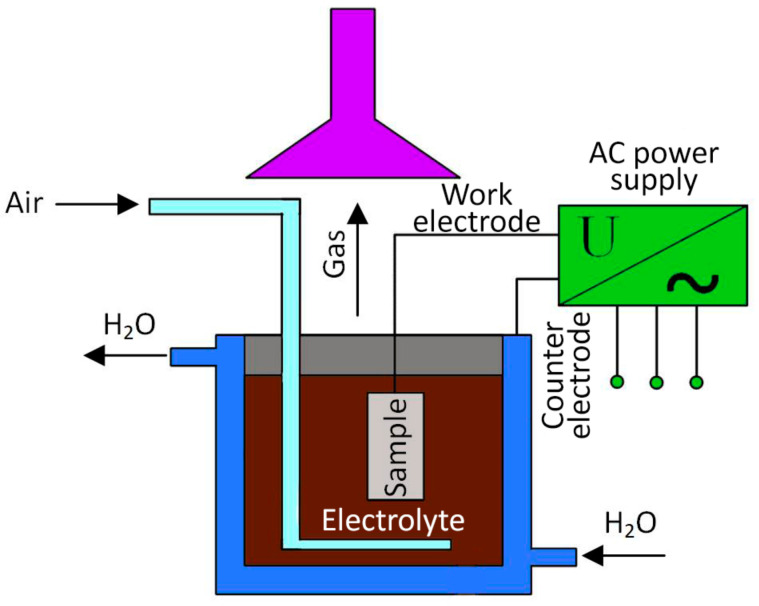
Schematic representation of the PEO process.

A schematic representation of the PEO process is shown in Figure 2. Ceramic coatings were applied to discs (with a 27 mm diameter and 7 mm thickness) from Ti6Al4V titanium alloy (Lkalloy, Shanghai, China); the structure and chemical composition are shown in Table 1. Before the PEO coating formation, the titanium samples were degreased with acetone for 25 min in an ultrasonic bath, washed with distilled water, and then cleaned by blowing with compressed air.

The PEO coatings were formed in the anode–cathode mode (50 Hz), with an anode-to-cathode current ratio of 1:1 and a sum current density of 20 A/dm^2^. The duration of the PEO treatment was 30 min. A silicate-hypophosphite base electrolyte consisting of 10 kg/m^3^ of Na_2_SiO_3_-9H_2_O and 5 kg/m^3^ of Na(PH_2_O_2_)-H_2_O was used with the addition of graphene oxide (0.1, 0.3, and 0.5 kg/m^3^). The electrolyte temperature was maintained at 25 °C; it was controlled in real-time using a resistance thermometer located in the treatment area of the samples. The electrolyte was stirred during PEO by blowing it with compressed air. To stabilize the GO particles, the suspensions were ultrasonically dispersed at 40 kHz for 20 min before each addition to the electrolyte. The pH and conductivity of the electrolytes were measured using a pH meter and conductivity meter (Mettler Toledo, Columbus, OH, USA). The sample codes and the composition of the electrolytes and their pH and conductivity are presented in Table 2.

**Figure 3:**
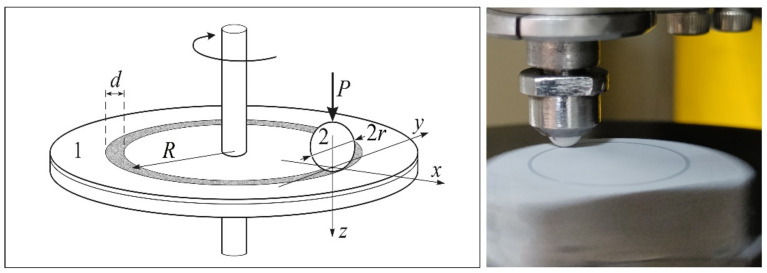
ASTM G-99 scheme of the friction contact (1—disk; 2—ceramic ball) (**left**); photo of rotating sample with PEO coating (**right**).

X-ray phase analysis was made in Cu Kα radiation at a voltage of 30 kV and angles of 2θ from 15 to 80 degrees on a diffractometer, a Difrey-401k (Scientific Instruments JSC, Saint Petersburg, Russia). Unidirectional ball-on-disk friction and wear tests were made using a UMT-3 (Bruker, Billerica, MA, USA) tribotester, according to the ASTM G99 standard [37] (rotation of the disk relative to the stationary ball pressed to it). The scheme of the friction contact and a photo of the rotating sample with the PEO coating are presented in Figure 3. Before testing, in order to remove all of the dirt from the surface of the sample and the ball, they were cleaned for 5 min in an ultrasonic bath with ethanol. The examined samples were not running in preliminary. A constant normal load, P = 5 N, was applied to an alumina (Al_2_O_3_) ball with a radius of r = 2 mm; the wear track nominal radius was R = 6 mm. The tests were performed at a sliding velocity of V = 0.1 m·s^−1^ (112.3 rpm). The acoustic emission from the contact was recorded during the tests. All tests were realized on a friction path, S = 1000 m, at room temperature, T = 23 °C, and relative humidity of 45…60%. Three tests were made for each sample code.

The sample volume losses, ΔV, were obtained by measuring the 3D surface profile of the wear tracks using a profilometer, a Talysurf CLI 500 (Taylor Hobson, Leicester, UK), which maps the measured area by tip–sample surface contact with a step and scanning velocity of 0.01 µm and 0.1 mm·s^−1^, respectively. In the case of a heterogeneous wear track, direct measurement of the wear volume using a profilometer is much more accurate than that recommended in ASTM G-99. The profilometer’s TalyMap Universal (Version 5.1) software allows one to select only the wear zone and calculate the entire volume of the material removed. The wear rate was calculated by the following relation:

W = ΔV/(P·S)(1)

Here, ΔV is the volume loss after the tests (mm^3^), P is the applied load, and (N) and S are the sliding distance (m). The morphology and surface structure of the PEO coatings were studied using a scanning electron microscope (SEM), a VEGA 3 LMH (Tescan, Brno, Czech Republic). Raman spectroscopy of the initial surface of PEO coatings and counter-bodies was made by DXR^TM2^, (Thermo Fisher Scientific, Waltham, MA, USA) using a 532 nm laser with a power of 2.0 mW.

## 3. Results and Discussion

**Figure 4:**
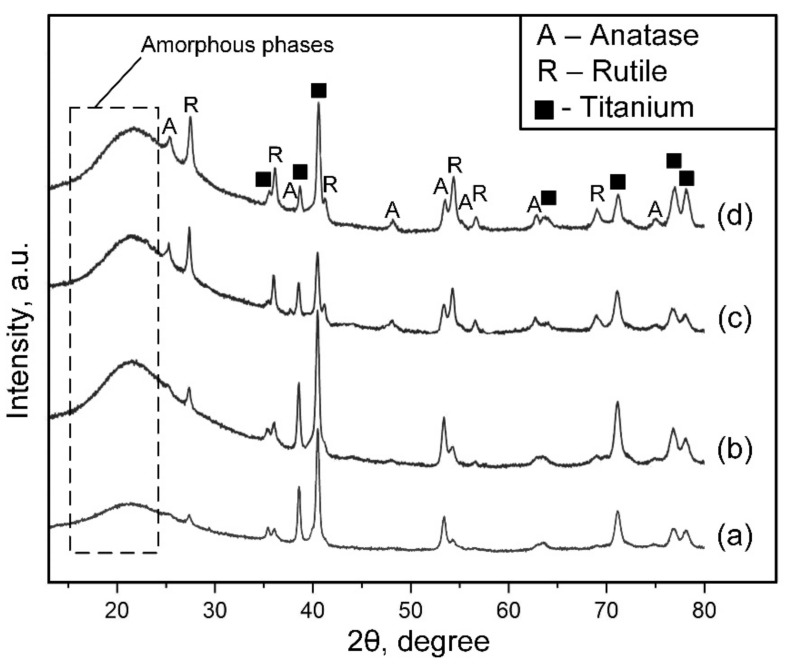
X-ray diffraction patterns of the PEO coatings on 0GO (**a**), 0.1GO (**b**), 0.3GO (**c**), and 0.5GO (**d**) sample surfaces.

The X-ray diffractogram (Figure 4) showed that the intensity of the rutile (the high-temperature TiO_2_ phase) peaks increased with the increasing GO concentration in the electrolyte. The growth of the peaks of the rutile phase is connected with the fact that the increasing GO concentration in electrolytes intensifies the PEO process due to a 1.5 times increase in the conductivity of the electrolytes (Table 2) and a consequent increase in the voltage drop and temperature in the discharges. As the PEO coating’s thickness increases, insufficient heating of the surface layer occurs, so anatase (the low-temperature TiO_2_ phase) is observed in the PEO coatings on the 0.3GO and 0.5GO sample surfaces.

**Figure 5:**
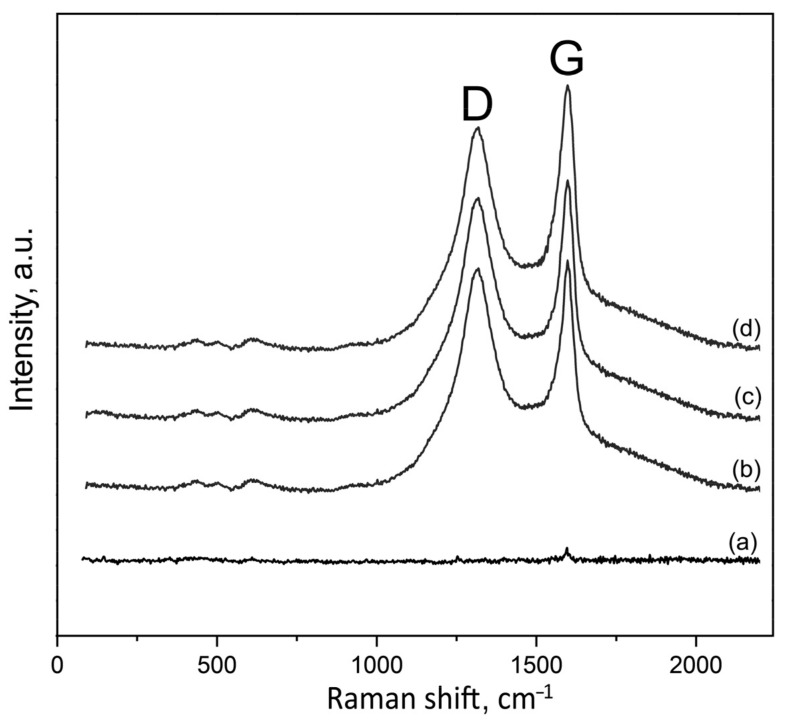
Raman spectra of the PEO coatings on 0GO (**a**), 0.1GO (**b**), 0.3GO (**c**), and 0.5GO (**d**) sample surfaces. “D” and “G” denote graphene oxide peaks.

Figure 5 shows the Raman spectra of the PEO coatings on the sample surfaces. The presence of GO in the PEO-coated samples, 0.1GO, 0.3GO, and 0.5GO, is confirmed by the detection of the characteristic D and G peaks of the graphene oxide (1356 cm^−1^ and 1597 cm^−1^, respectively). No increase in the D and G peak intensities with the increase of the GO concentration in the electrolyte from 0.1 to 0.5 kg/m^3^ was observed, which was probably due to the fact that in the PEO process, some of the GO particles coagulated and did not take part in the coating formation.

**Figure 6:**
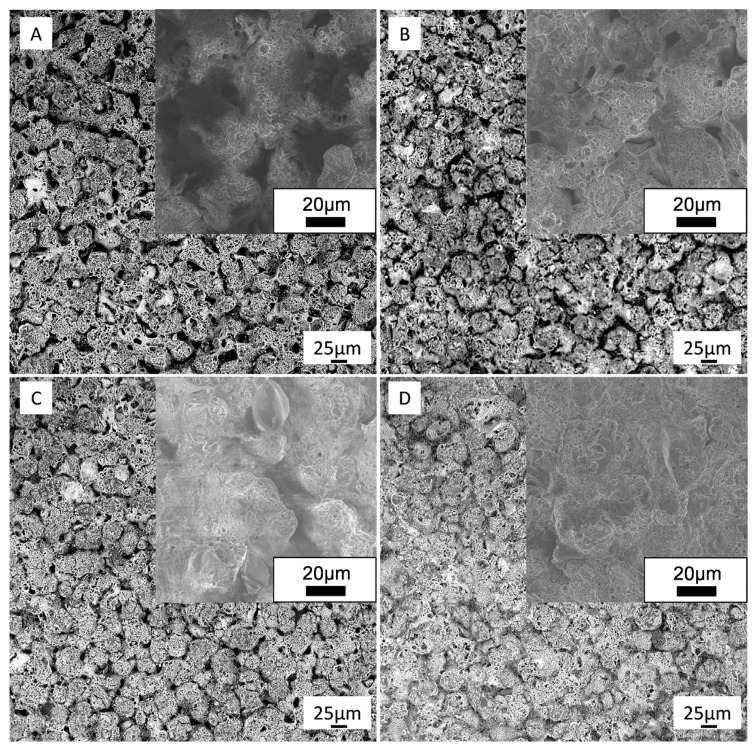
SEM images (general view and close-up) of the surface morphology of different PEO-coated samples: 0GO (**A**), 0.1GO (**B**), 0.3GO (**C**), and 0.5GO (**D**).

Figure 6 shows the surface morphology of the PEO coatings formed in the electrolytes with different GO contents. As can be seen, the surface of the coatings contains many micropores and microspheres formed by the ejection of molten material from the discharge channels during the PEO treatment. As the GO concentration increased, the number and diameter of pores decreased due to their filling with the GO particles.

**Figure 7:**
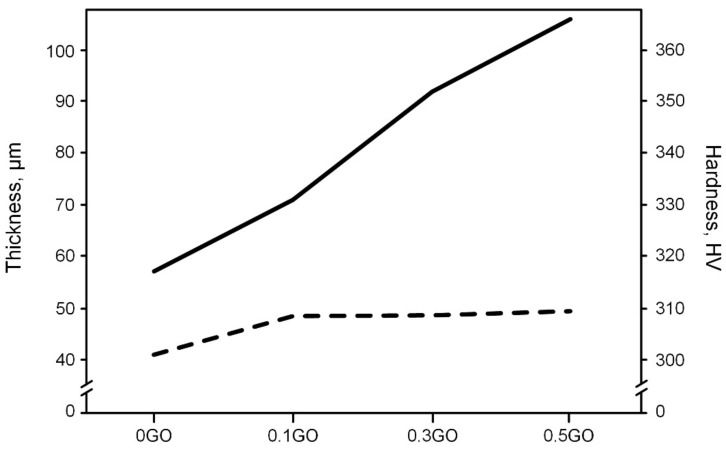
PEO coatings’ thickness (**- - -**) and hardness (**―**), depending on the GO concentration in the electrolytes.

Figure 7 shows the effect of the GO concentration in the electrolytes on the PEO coatings’ thickness and Vickers’ hardness.

As can be concluded, the increase in the coating thickness of the 0.1GO sample compared to the 0GO sample was approximately 18.5%. A further increase in the concentration of GO in the electrolyte had no significant effect on the growth of the PEO coatings’ thickness. The reduction of the coating’s growth rate of the samples 0.3GO and 0.5GO may be due to the blocking of the discharge channels or coagulation of graphene oxide particles during the PEO process at excessive amounts of GO [38]. The addition of GO to the base electrolyte resulted in a significant increase in the hardness of the PEO coatings. The maximum hardness (366 HV) was demonstrated by PEO coatings formed in the electrolyte with 0.5 kg/m^3^ of graphene oxide. The increase in the hardness of the PEO coatings with an increasing GO concentration in the electrolyte is due to the formation of a composite structure, which includes titanium carbide [39]. It is also worth noting that a similar increase in the hardness of the coatings was obtained by Y. Zuo et al. [38].

**Figure 8:**
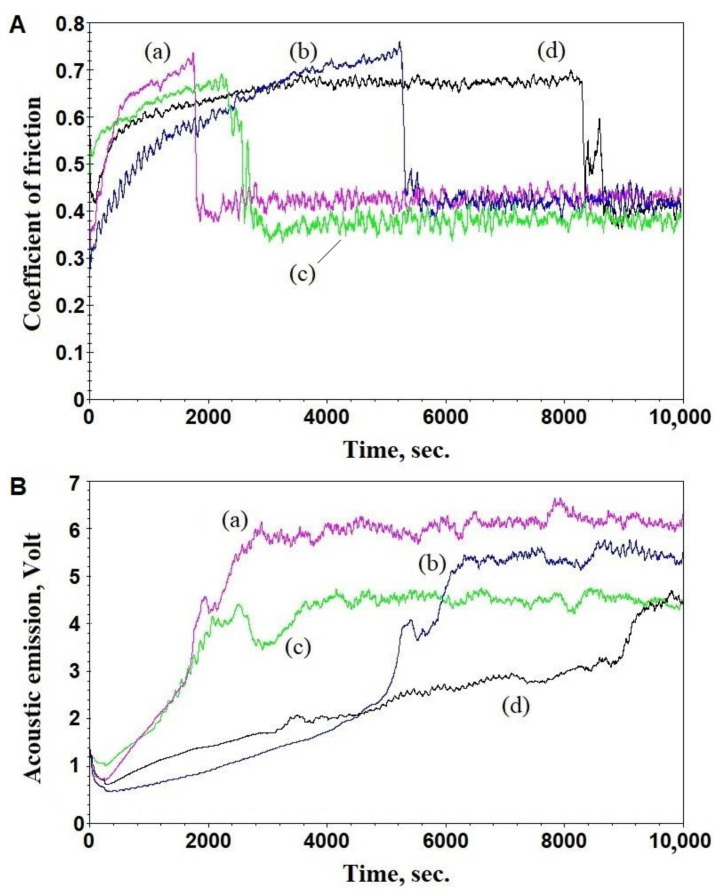
Dependence of the coefficient of friction (**A**) and acoustic emission (**B**) on the test time for samples 0GO (**a**), 0.1GO (**b**), 0.3GO (**c**), and 0.5GO (**d**).

Figure 8A shows the dependences of the friction coefficient (the COF) on the test time obtained at P = 5 N, V = 0.1 m·s^−1^, and S = 1000 m. An analysis of the results shows that the COF for the samples 0GO, 0.1GO, and 0.3GO did not stabilize during the test, so the value of the COF was fixed at the moment of coating failure (delamination). Delamination is characterized by the sharp decrease of the COF (Figure 8B), as well as by the changes in the acoustic emission (the monotonic increase in the emission is replaced by a local decrease in the signal when the coating is detached from the substrate, followed then by signal oscillations relative to some average value, which are specific for wear process). For the 0.5GO sample, the coefficient of friction stabilized during the test, so we had a steady-state COF value. After the coating delamination, the value of the COF corresponded to the Al_2_O_3_-bare Ti6Al4V frictional contact. Delamination of coatings during wear occurs due to contact fatigue, and with an increase in the GO concentration in the electrolyte from 0 to 0.5 kg/m^3^, this process slows down by a multiple from less than 2000 to more than 8000 s.

**Figure 9:**
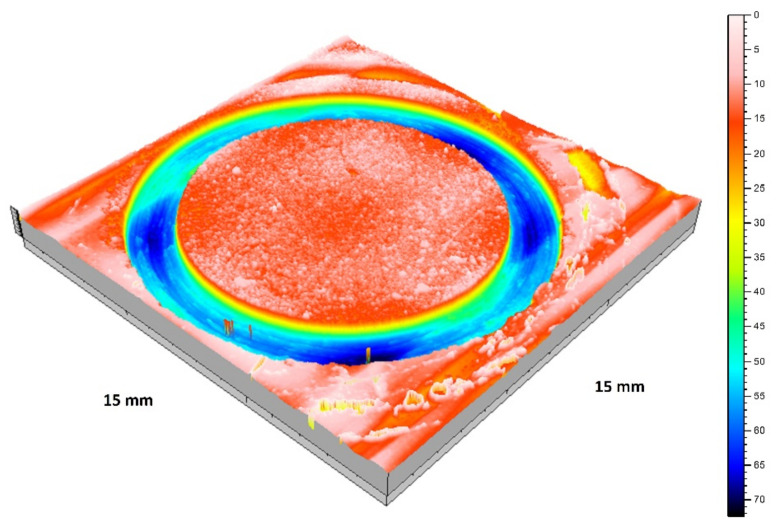
Representative 3D wear track topography corresponding to 0.5GO sample as a function of depth in µm (colored scale).

The representative 3D wear track topography for the 0.5GO sample after sliding against the alumina ball is presented in Figure 9.

From the obtained 3D wear track surface topographies, the volume loss was calculated to determine the wear rate using Equation (1). The coefficient of friction and the wear rate for all the samples are presented in Table 3. The analysis shows that the PEO coatings formed in the electrolytes with GO additives showed a slight decrease in the COF, from 0.73 to 0.69, and a significant decrease in the wear rate, from 8.04 to 5.2 mm^3^/N·m, with an increase in the GO concentration in the electrolyte, from 0 to 0.5 kg/m^3^, which were presented in the SEM images (general view and close-up) of the surface morphology of different PEO-coated samples: 0GO (A), 0.1GO (B), 0.3GO (C), and 0.5GO (D). It is important, that the wear is measured after the coating delamination, including the wear of bare Ti6Al4V (according to the ASTM G-99 standard regulating the length of the friction path, 1000 m, or the test time 10,000 s, at a sliding velocity of 0.1 m·s^−1^). Our results are comparable with the results obtained in previously published works. For example, in a recently published work [40], the coefficient of friction in the dry sliding wear tests of the coatings against a steel ball (100 Cr6) using a load of 5 N is an average value of ~0.8 compared with ~0.6 for the untreated titanium. Even if the COF values are higher for the PEO-treated samples, the estimated wear rates are 50% lower compared to the titanium substrate.

**Figure 10:**
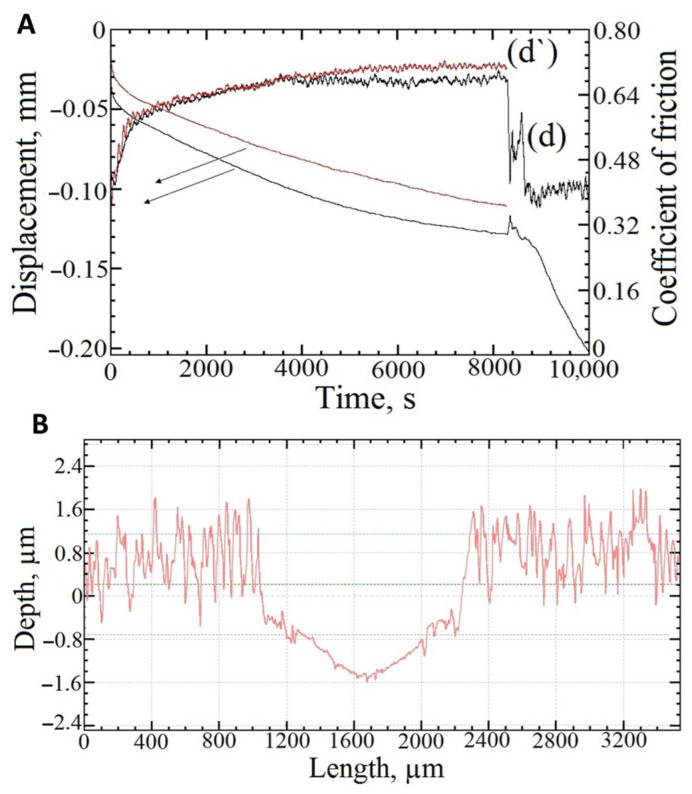
The experimental results for the 0.5GO samples: displacement due to wear and the COF vs. test time, where curves d and d’ are obtained for full and ‘short’ tests, respectively (**A**); the profile of the wear track after the ‘short’ test (**B**).

A ‘short’ test, stopped at the approximate moment of the coating failure, was made for the most wear-resistant sample (0.5GO) (Figure 10). The motivation was to evaluate the wear of the coating material itself. Measurement of the friction path showed that the coating failed instantaneously at the substrate–coating interface due to the contact fatigue; the coating wear at the end of the ‘short’ test was small (Figure 10B). From the analysis of the acoustic emission, it was concluded that the emission decreases with the addition of the graphene oxide in the electrolyte and has the character of a gradual increase in the time for all of the coatings, which indicates the gradual accumulation of micro-defects, maybe in the coating–substrate interface.

Thus, during the wear of the samples with PEO coatings formed in the electrolytes with GO additives, the surface is smoothed as a result of the wear of the coating asperities. At the end of this stage, the wear process is slow, but the delamination of the coating occurs from the interface with the substrate (the Ti6Al4V alloy). The time to delamination depends on the test conditions and the GO concentration in the electrolyte. Thus, the most likely cause of the fracture is the development of a fatigue crack at the interface coating–substrate with its subsequent emergence to the surface. To analyze this phenomenon, we calculated the stresses that occurred in the 0.5GO sample at time *t_0_*, when the surface was smoothed, and the ball wear was negligible, and also at the final stage of the ‘short’ testing, when the ball wear significantly changed the type of contact. The choice of the sample was due to the longest time until the coating fracture. The formulation of the elastic contact problem and a brief description of the numerical-analytical method of the solution are given in Appendix A. The following input parameters were used for the calculation: the modulus of elasticity of the coating and substrate, 350 GPa and 110 GPa, respectively, Poisson’s ratios (0.25 and 0.33), the coating’s thickness after running in the rough layer (20 µm), the load, 5 N, the COF 0.6, the ball radius, 2 mm, and the modulus of elasticity and Poisson’s ratio of the ball material (Al_2_O_3_), 380 GPa and 0.25, respectively. The worn spot on the surface of the ball has a shape close to circular, with an area of 1.4 mm^2^. It was assumed that a constant pressure of 3.5 MPa occurs on this spot, and along the edges of the contact zone, the pressure rapidly tends to zero according to parabolic law.

**Figure 11:**
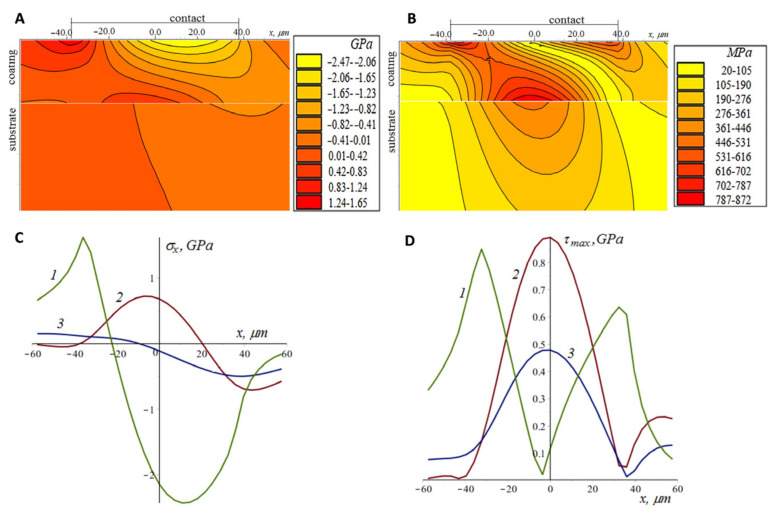
Tensile–compressive (**A**,**C**) and principle shear (**B**,**D**) stresses in the coating and the substrate (contact with the unworn ball) at the surface (curves 1), interface for coating material (curves 2), and interface for substrate material (curves 3).

**Figure 12:**
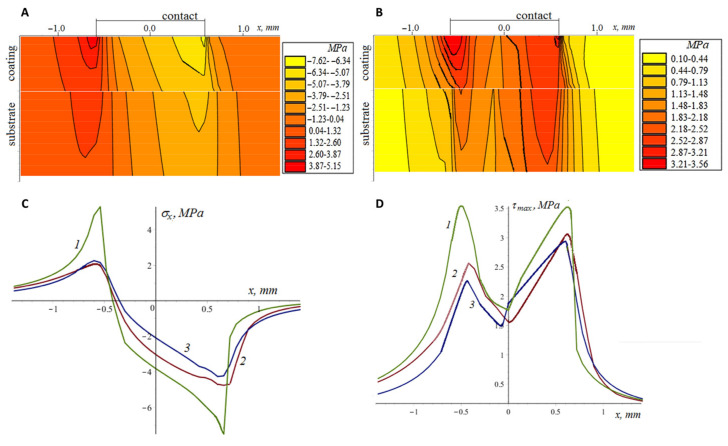
Tensile–compressive (**A**,**C**) and principle shear (**B**,**D**) stresses in the coating and the substrate (contact with the worn ball) at the surface (curves 1), interface for coating material (curves 2), and interface for substrate material (curves 3).

Tensile–compressive and principal shear stress distributions in the coating and the substrate were analyzed. Concentrations of tensile stresses can lead to brittle failure, and these stresses contribute to the opening and development of existing macro- and microcracks. Principal shear stresses are often used in the criteria of plastic deformation, as well as in the criteria of damage accumulation, including the accumulation process at the coating–substrate interface [41,42]. The stresses shown in Figure 11 and Figure 12 were obtained in a plane parallel to the sliding direction (sliding along the Ox axis) and passing through the center of the contact area. The stresses are considered to have a discontinuity at the interface due to the difference in elastic properties and the equal displacements of the materials at the interface. The maximum contact pressure obtained by solving the contact problem for the ball and the coated elastic material is 0.95 GPa. At the initial stage (Figure 11), the coating thickness is comparable to the size of the contact spot. The maximum tensile stress occurs on the surface behind the contact spot (more precisely, on its boundary); with depth, the tensile stresses decrease sharply. The maximum tensile stresses at the interface are located almost under the center of the contact spot and are due to the bending of the coating. The principal shear stresses at the interface are quite high (0.87 GPa in the coating material and 0.48 GPa in the substrate material), which can lead to a rapid accumulation of fatigue damage at the interface under cyclic loading and unloading. At the same time, the configuration of tensile and compressive stresses does not contribute to the appearance of the vertical cracks passing through the coating. During the tests, ball wear occurs (Figure 12), resulting in an increase in the size of the contact spot and a decrease in maximum pressure (more than 25 times at the end of the test). Thus, there is a decrease in the damage accumulation rate. At the same time, the relative thickness of the coating decreases, the stresses become more homogeneous over the coating’s thickness, and the zones of tension and compression passing from the surface to the interface contribute to the development of vertical cracks and the coating delamination.

**Figure 13:**
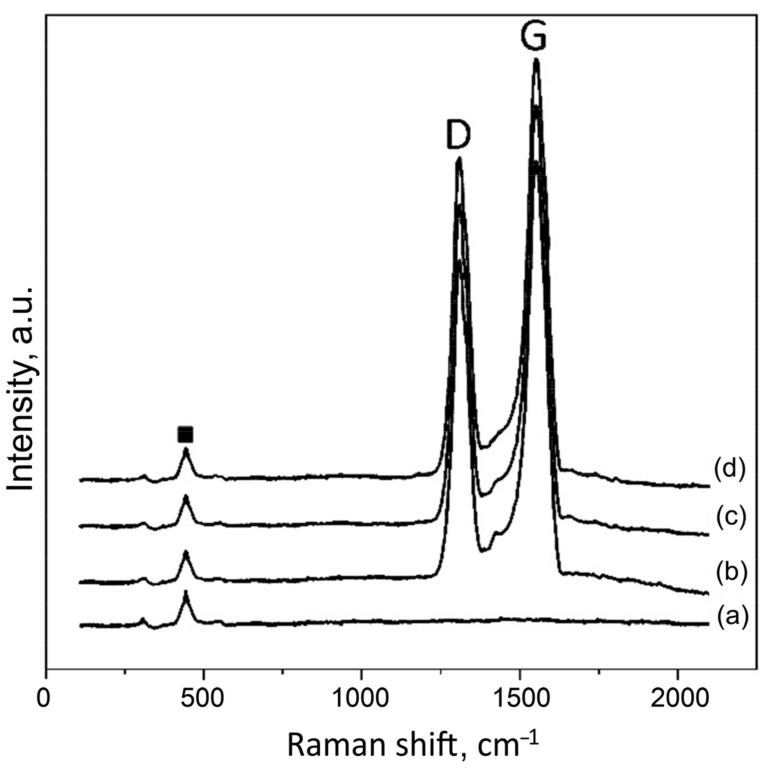
Raman spectroscopy of the worn surface of alumina balls after tribological tests of 0GO (**a**), 0.1GO (**b**), 0.3GO (**c**), and 0.5GO (**d**) samples, where “D” and “G” are graphene oxide peaks, and “▪”is the—Al_2_O_3_ peak.

The presence of graphitic structures protects the surface during tribotests due to the effect of self-lubrication. Figure 13 shows the Raman spectroscopy of the alumina ball after the tribological tests, with GO peaks, which means that these graphitic structures transfer to the counter-body surface.

**Figure 14:**
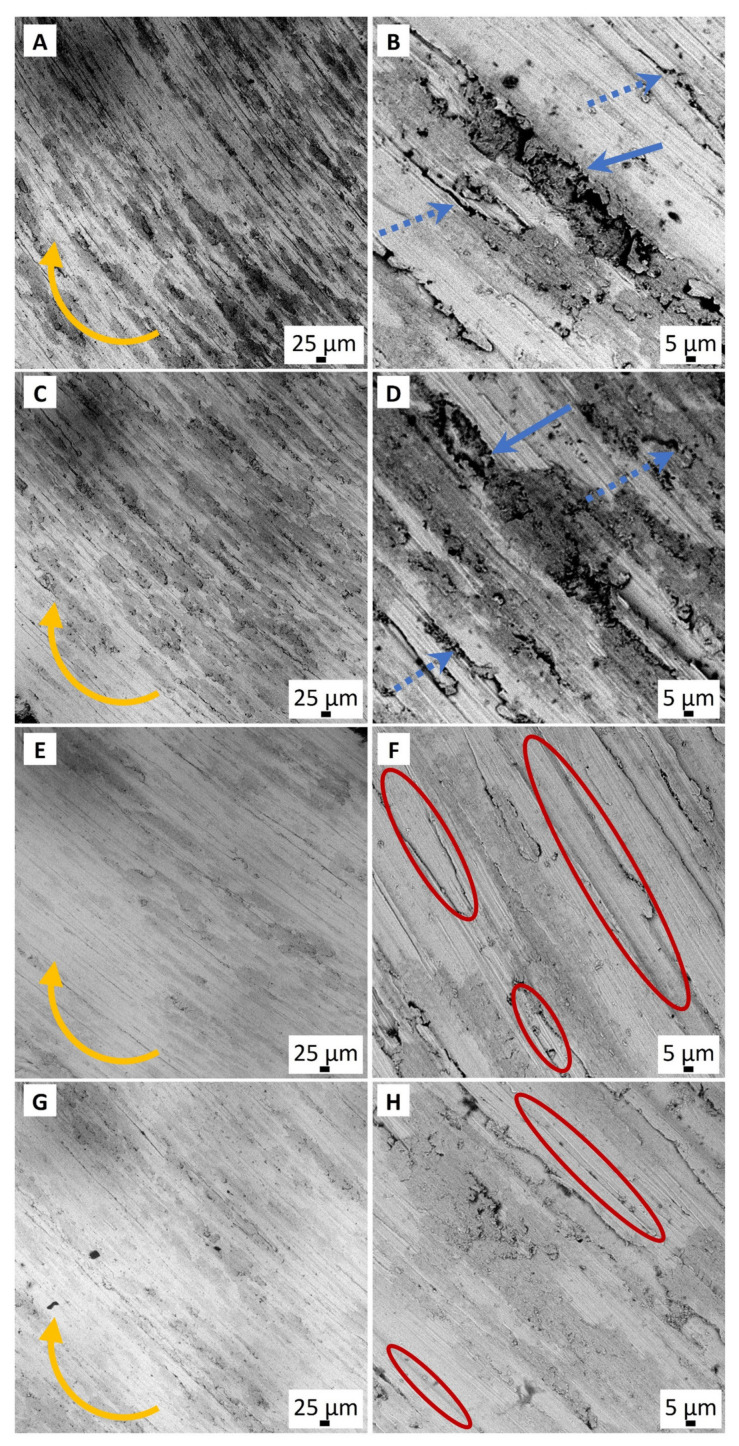
SEM micrographs of the worn surface of 0GO (**A**,**B**), 0.1GO (**C**,**D**), 0.3GO (**E**,**F**), and 0.5GO (**G**,**H**) samples after sliding against alumina ball. Yellow, blue, and blue-dotted arrows show the rotation direction, evidence of pull-out, and cracks, respectively. The grooves on the wear scars are marked with a red oval.

The effect of the lubricating layer on the wear process can be analyzed from SEM micrographs of the worn surfaces (Figure 14). It is important that the coatings are delaminated during the wear tests, but GO-containing structures exist at the counter-body and Ti6Al4V alloy surfaces, except in the 0GO sample. The morphology of the wear tracks of the 0.5GO and 0.3GO samples is smoother than for the 0GO and 0.1GO samples.

## 4. Conclusions

The synergistic effect of adding graphene oxide to the base electrolyte and PEO treatment of Ti6Al4V alloys can improve the properties of the latter. With an increase in the content of graphene oxide in the electrolyte from 0 to 0.1 kg/m^3^, the thickness of the coatings increases by about 20%, and with a further increase in the concentration of GO from 0.1 to 0.5 kg/m^3^, a very slight increase in the thickness of the coatings occurs. The input of graphene oxide additives into the base silicate-hypophosphite electrolyte leads to an increase in the hardness of PEO coatings from 331 to 366 HV with an increase in the GO concentration from 0.1 to 0.5 kg/m^3^. The friction coefficient and wear rate of the coatings after PEO treatment decrease from 0.73 to 0.69 and from 8.04 to 5.2 mm^3^/N·m when the GO concentration in the electrolyte increases from 0 to 0.5 kg/m^3^. This occurs due to the formation of a GO-containing lubricating tribolayer upon contact with the coating and the counter-body in the friction pair, which is confirmed by the Raman spectroscopy of their worn surfaces. Delamination of the coatings during wear occurs due to contact fatigue, and with an increase in the GO concentration in the electrolyte, this process slows down by a multiple from less than 2000 to more than 8000 s.

## Figures and Tables

**Table 1:** Structure and chemical composition of Ti6Al4V titanium alloy [36].

Structure	Chemical Composition, wt.%							
α + β	Al	V	C	H	N	Fe	O	Ti
	5.5–6.75	3.5–4.5	0.08	0.015	0.05	0.3	0.2	Bal

**Table 2:** The composition of electrolytes, their pH and conductivity, and sample codes.

Sample Codes	Base Electrolyte	Graphene Oxide Addition, kg/m^3^	pH	Conductivity, mS∙cm^−1^
0GO	Na_2_SiO_3_∙9H_2_O + Na(PH_2_O_2_)∙H_2_O	0	12.25	31.23
0.1GO		0.1	12.21	37.87
0.3GO		0.3	12.12	41.34
0.5GO		0.5	12.04	47.61

**Table 3:** The tribological tests results.

Samples Code	Coefficient of Friction	Wear Rate, mm^3^/N·m
0GO	0.73 ± 0.02	(8.04 ± 0.05)·10^−4^
0.1GO	0.72 ± 0.02	(6.77 ± 0.04)·10^−4^
0.3GO	0.70 ± 0.01	(5.86 ± 0.03)·10^−4^
0.5GO	0.69 ± 0.01	(5.2 ± 0.03)·10^−4^

## Data Availability

Not applicable.

## Appendix A

This appendix presents mathematical formulations of problems for determining the contact and internal stresses in an elastic coating and an elastic substrate, as well as at their interface. A simplifying assumption was made that the solution to the contact problem does not depend on shear stresses due to friction. This allows one to solve the contact problem separately and then calculate the stresses by introducing friction according to the Amonton–Coulomb law.

In the first stage, the contact problem was solved for a spherical indenter and a two-layer elastic half-space. A normal force, P, directed along its axis of symmetry acts upon the indenter. Friction forces are absent. The problem is considered in an axisymmetric formulation. In a cylindrical coordinate system associated with the point of initial contact, the boundary conditions on the surface of the half-space are the following:

(A1) $$\begin{array}{l} w^{\left(1\right)}+w^{\left(0\right)}=f(r)+\delta ,\;0\leq r\leq a;\\ \sigma_{z}^{\left(1\right)}=0,\;\;\;\;\;\;a<r<\infty ;\\ \tau_{xz}^{\left(1\right)}=\tau_{yz}^{\left(1\right)}=0\\ r=\sqrt{x^{2}+y^{2}} \end{array}$$

Here, $w^{\left(1\right)}$ and $w^{\left(0\right)}$ are the elastic displacements of the surfaces of the layer and the indenter, respectively; δ is the approach of the two bodies; a is the radius of the contact area, which is an unknown value for smooth indenters. The equilibrium condition is also satisfied:

(A2) $$P=\int_{0}^{2\pi }\int_{0}^{a}p(r)drd\varphi ,\;p(r)=\sigma_{z}^{\left(1\right)}(r,0)\;\;r\leq a$$

Here, *p(r)* is the contact pressure. For a smooth indenter, the condition of zero-normal stresses at the boundaries of the contact area is also taken into account:

(A3) $$\sigma_{z}^{\left(1\right)}(r)=0,\;\;r=a$$

Conditions at the coating–substrate interface are the following:

(A4) $$\begin{array}{l} w^{\left(1\right)}=w^{\left(2\right)},\;\sigma_{z}^{\left(1\right)}=\sigma_{z}^{\left(2\right)},\\ u_{x}^{\left(1\right)}=u_{x}^{\left(2\right)},\;u_{y}^{\left(1\right)}=u_{y}^{\left(2\right)},\;\tau_{xz}^{\left(1\right)}=\tau_{xz}^{\left(2\right)},\;\tau_{yz}^{\left(1\right)}=\tau_{yz}^{\left(2\right)} \end{array}$$

$w^{\left(i\right)}$ and $u_{i}^{\left(i\right)},\;u_{i}^{\left(i\right)}$ are normal and tangential displacements in the coating (*i* = 1) and the substrate (*i* = 2).

The problem with boundary conditions (A1)–(A4) is solved using a method based on Hankel integral transformations, the boundary element method, and an iterative procedure [43]. The radius of the contact area and the distribution of the contact pressure are then determined.

At the next stage, internal stresses are calculated based on the Amonton–Coulomb law:

(A5) $$\begin{array}{l} \tau_{xz}^{\left(1\right)}=\mu p(r),\;0\leq r\leq a;\\ \tau_{xz}^{\left(1\right)}=0,\;\;\;a<r<\infty ;\\ \tau_{yz}^{\left(1\right)}=0 \end{array}$$

The method for calculating stresses in a two-layered elastic half-space is based on double integral Fourier transforms [44].

When the ball wears out, on the worn spot, Ω, a constant pressure, $p_{0}$, acts. Outside the Ω, it quickly goes to zero (zone Ω′). The boundary conditions (A1) are changed to:

(A6) $$\begin{array}{l} \sigma_{z}^{\left(1\right)}=p_{0},\;(x,y)\in \Omega ;\\ w^{\left(1\right)}+w^{\left(0\right)}=f(x,y)+\delta ,\;(x,y)\in \Omega \prime ;\\ \sigma_{z}^{\left(1\right)}=0,\;\;(x,y)\notin \Omega ,\;(x,y)\notin \Omega \prime ;\\ \tau_{xz}^{\left(1\right)}=\tau_{yz}^{\left(1\right)}=0 \end{array}$$

Conditions (A2)–(A6) are used to determine stresses under the worn ball. The contact problem is not axisymmetric, and for its solution, the method presented in [45] was used.
